# The usefulness of wire-guided endoscopic snare papillectomy for tumors of the major duodenal papilla

**DOI:** 10.1371/journal.pone.0211019

**Published:** 2019-01-23

**Authors:** Masanori Kobayashi, Shomei Ryozawa, Hirotoshi Iwano, Ryuichiro Araki, Yuki Tanisaka, Akashi Fujita, Tsutomu Kobatake

**Affiliations:** 1 Department of Gastroenterology, Saitama Medical University International Medical Center, Hidaka-City, Saitama, Japan; 2 Gastroenterology and Endoscopy Center, Shibetsu City Hospital, Shibetsu-City, Hokkaido, Japan; 3 Community Health Science Center, Saitama Medical University, Moroyama-machi, Iruma-gun, Saitama, Japan; Hvidovre Hospital, DENMARK

## Abstract

**Objectives:**

Although endoscopic papillectomy is useful for treating papillary tumors, it is associated with a high rate of complications including pancreatitis; therefore, safer treatment options are needed. We examined the utility of wire-guided endoscopic papillectomy by comparing the pancreatic duct stenting and pancreatitis rates before and after wire-guided endoscopic papillectomy was introduced at our institution.

**Methods:**

We retrospectively examined the data from 16 consecutive patients who underwent conventional endoscopic papillectomy between November 1995 and July 2005 and the data from 33 patients in whom wire-guided endoscopic papillectomy was first attempted at our institution between August 2005 and April 2017. We compared the pancreatic duct stenting and pancreatitis rates between the two groups.

**Results:**

Of the 33 patients in whom wire-guided endoscopic papillectomy was first attempted, the procedure was completed in 21. Pancreatic duct stenting was possible in 30 of the 33 patients in whom wire-guided endoscopic papillectomy was attempted (91%), and this rate was significantly higher than that before the introduction of wire-guided endoscopic papillectomy (68.8%). The incidence of pancreatitis before the introduction of wire-guided endoscopic papillectomy was 12.5%, but after August 2005, the incidence was reduced by half to 6.1%, which includes those patients in whom wire-guided endoscopic papillectomy could not be completed.

**Conclusions:**

Although wire-guided endoscopic papillectomy cannot be completed in some patients, we believe that this method shows some potential for reducing the total incidence of post-endoscopic papillectomy pancreatitis owing to more successful pancreatic duct stenting.

## Introduction

Tumors of the major duodenal papilla are relatively rare, with most of these tumors being adenoma or carcinoma. For patients with carcinoma, surgical pancreatoduodenectomy is considered an appropriate treatment [1]. In contrast, for patients with localized adenoma of the papilla, no treatment guidelines have been defined [2]. Although many reports on the usefulness of endoscopic papillectomy (EP) for treating localized adenoma of the papilla exist [3–8], EP is associated with many complications. For instance, the American Society for Gastrointestinal Endoscopy Guidelines mention pancreatitis, perforation, bleeding, and cholangitis as acute-phase complications [2]. Of these, acute pancreatitis is the most frequent and potentially lethal complication, although it can be prevented, unlike perforation and bleeding. Thus, treatments for or methods of preventing EP-associated acute pancreatitis are needed.

It has previously been reported that pancreatic duct stenting is effective for treating post-endoscopic retrograde cholangiopancreatography pancreatitis [9]. Pancreatitis after EP can also be prevented with pancreatic stents [10,11]. However, after resection of the papilla, pancreatic duct cannulation often becomes very difficult owing to the bleeding and edema that are caused by electrocautery. Therefore, a reliable method of pancreatic duct stenting is required.

In 2005, Moon et al. [12] proposed pancreatic duct wire-guided EP (WGEP). Currently, however, the utility of this method for reducing the incidence of EP-related pancreatitis remains unknown. In the present study, we examined the usefulness of WGEP by comparing the rates of pancreatic duct stenting and pancreatitis before and after the introduction of WGEP at our institution.

## Methods

This study was approved by the Saitama Medical University International Medical Center institutional review board (Approval No. 17-041) and conformed to the principles of the Declaration of Helsinki (as revised in Fortaleza, Brazil, in October 2013). All patients’ data were completely anonymized before we accessed them. As per the directives of the institutional review board, although the requirement for informed consent was waived, we ensured that the information about this study was released to the public on our homepage and guaranteed the subjects of this study an opportunity of refusal to participate in the study.

### Patients and EP procedures

We collected data from consecutive patients who initially underwent EP for tumors of the major duodenal papilla between November 1995 and April 2017 under the care of S.R. and H.I., who were EP experts as of 1995. Between November 1995 and July 2005, 16 patients underwent conventional EP. In conventional EP, the tumor was grasped and resected with an electrosurgical snare. Snare resection was performed in a radical fashion. Lesions that could not be resected en bloc were resected in a piecemeal fashion. After the bile and pancreatic ducts were identified with the sphincter of Oddi at the resected surface, a standard cannula (MTW Endoskopie, Wessel, Germany) was inserted into the pancreatic duct and a 0.035-inch guidewire was placed (Jagwire, Boston Scientific, Natick, MA, USA) as often as possible. Subsequently, another guidewire was placed in the bile duct in the same fashion, if possible. A 7-Fr biliary stent (QuickPlaceV, Olympus Corp., Tokyo, Japan) was then placed, followed by a 5-Fr double-flap pancreatic stent (Geenen, Cook Medical Inc., Bloomington, IN, USA). This order of stent insertion can prevent migration of the pancreatic duct stent.

**Fig 1:**
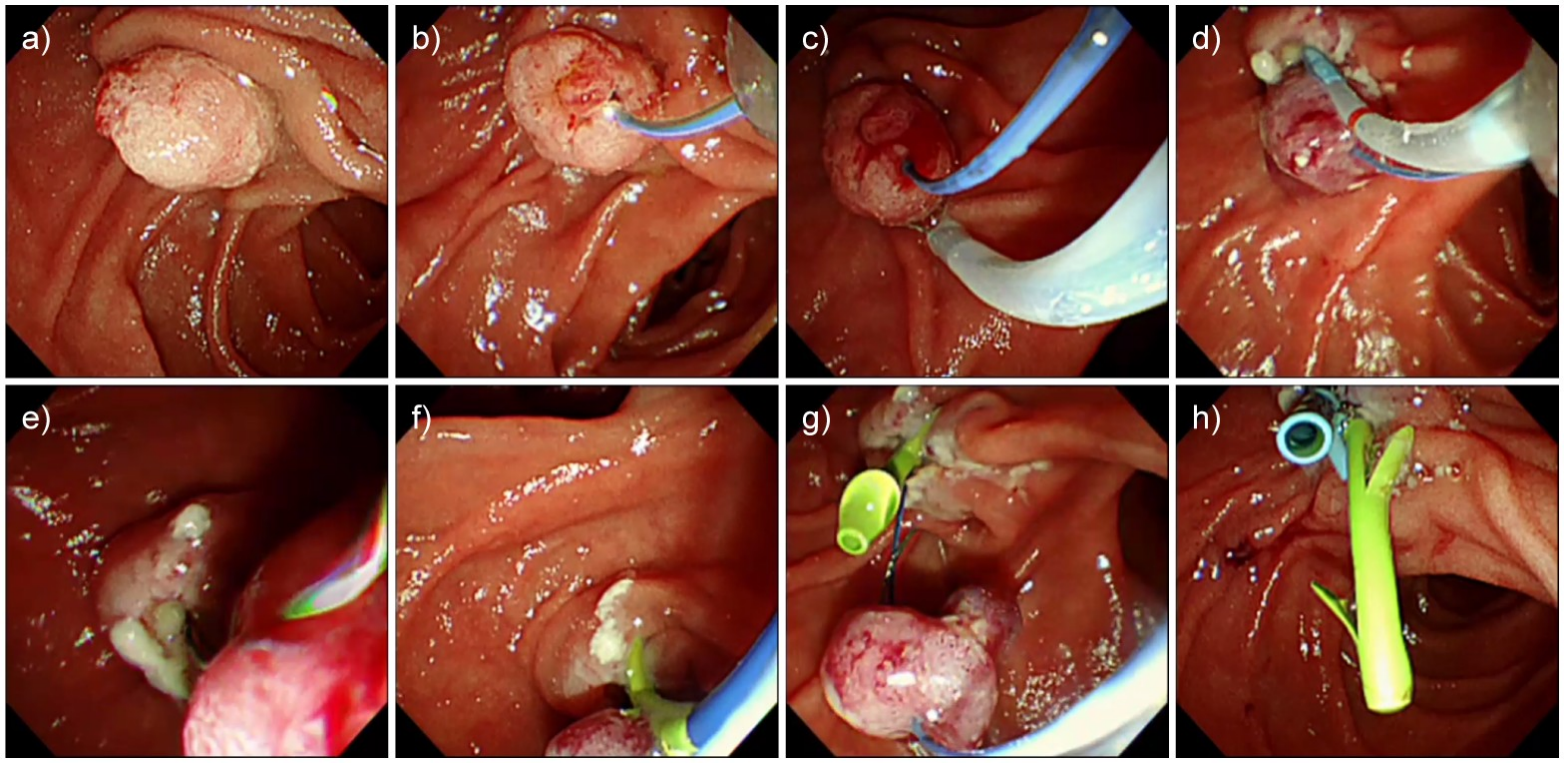
Pancreatic duct wire-guided endoscopic papillectomy. a) Tumor of the papilla prior to the procedure. b) The pancreatic duct was cannulated, and an insulated guidewire was placed. c) An electrosurgical snare was inserted in a monorail fashion over the insulated guidewire, and then the tumor of the papilla was grasped and resected. d) The snare was removed, leaving the specimen intact, and the pancreatic duct was re-cannulated beside the insulated guidewire. e) A normal guidewire was placed into the pancreatic duct. f) A pancreatic stent was placed through this guidewire. g) A snare was inserted in a monorail fashion over the insulated guidewire, and the tip of the guidewire was grasped at the distal end of the specimen. The specimen was then collected with a scope. h) The bile duct was cannulated, and a biliary stent was placed.

In August 2005, we began attempting WGEP; thus, data from the 33 patients in whom WGEP was attempted from August 2005 to April 2017 were included. We performed WGEP according to the procedures described below and depicted in Fig 1 and S1 Video. First, the pancreatic duct was cannulated and an insulated 0.018-inch guidewire (MTW Endoskopie) was placed (Fig 1b). Second, an electrosurgical snare was inserted in a monorail fashion over the insulated guidewire, and the tumor of the papilla was grasped and resected (Fig 1c). Third, the snare was removed, leaving the specimen intact, and the pancreatic duct was re-cannulated beside the insulated guidewire (Fig 1d). Fourth, a normal guidewire (VisiGlide2, Olympus Corp.) was placed into the pancreatic duct (Fig 1e), and a 5-Fr double-flap pancreatic stent (Geenen, Cook Medical Inc.) was placed through this guidewire (Fig 1f). Following this, a snare was inserted in a monorail fashion over the insulated guidewire, the tip of the guidewire was grasped at the distal end of the specimen, and the specimen was collected with a scope (Fig 1g). Finally, if possible, the bile duct was cannulated, and a 7-Fr biliary stent (QuickPlaceV, Olympus Corp.) was placed (Fig 1h). If any of the first three steps could not be completed, papillectomy was performed by switching to the conventional EP method.

In the original procedures for WGEP, a 0.035-inch guidewire was used to preserve the pancreatic duct, and the pancreatic stent was placed using this guidewire [12]; however, here, we used a 0.018-inch isolated guidewire because it is easier to handle and manipulate. Despite complicating the procedure slightly, the use of this guidewire allows the tumor to be easily grasped by the snare and resected safely.

For these procedures, we used either a TJF240 or TJF260V duodenoscope (Olympus Corp.). Between 1995 and 1999, an electrosurgical snare (SD-9/12U-1, Olympus Corp.) was used. Thereafter, the SnareMaster snare (SD-210U-10/15/25, Olympus Corp.) was used. The ICC200 electrosurgical unit (Erbe Elektromedizin, Tubingen, Germany) was used for resection until August 2013, and the mechanical settings were the “Endocut” mode, set at “effect3 (Cut 120 W/Coagulation 30 W).” However, beginning in September 2013, the ESG-100 electrosurgical unit (Olympus Corp.) was used, and its mechanical settings were “Pulse cut slow (Level 30).”

After undergoing EP, patients were prescribed antibiotics and nafamostat mesylate or gabexate mesylate preventively. No rectal nonsteroidal anti-inflammatory drugs were administered, regardless of the results of pancreatic duct stenting.

### Data collection and assessments

The following data were collected from patients’ medical records: patient age, sex, final histological diagnosis, tumor size, success or failure of en bloc resection, success or failure of pancreatic duct stenting, and the presence or absence of procedural complications such as pancreatitis, bleeding, and cholangitis after EP. We also examined whether patients subsequently required additional therapy. When the histological diagnosis was invasive adenocarcinoma, we recommended pancreatoduodenectomy. If we found tumor recurrence after at least 1 year of follow-up, we performed EP or argon plasma coagulation (APC), depending on the situation. Moreover, we calculated the incidence of pancreatitis in each patient group. The severity of complications was assessed according to the Cotton classification [13].

### Statistical analysis

Statistical analyses were performed using the SAS JMP 13.0.0 statistical software (SAS Institute, Inc., Cary, NC, USA). The sex distributions, rate of en bloc resection, rate of each additional therapy, rate of pancreatic duct stenting, and incidence of post-EP pancreatitis, cholangitis, and bleeding between the two patient groups (before and after the introduction of WGEP) were compared with Fisher’s exact tests. The Shapiro-Wilk normality test was used to confirm the normality of age and tumor size, and these were compared between the groups with Mann-Whitney U tests. The proportions of the final histological diagnoses were compared with the Fisher-Freeman-Halton test. Differences were considered statistically significant at P <0.05.

## Results

A total of 49 patients were included in this study. The median age of the patients was 65 years (interquartile range: 59–70 years) and the median tumor diameter was 11 mm (interquartile range: 10–20 mm). Regarding the final histological diagnosis, 33 patients had an adenoma, 14 had carcinoma including carcinoma-in-adenoma, 1 had hyperplasia, and 1 had no evidence of tumor tissue on resection. No significant differences in these factors were noted between the two patient groups (before and after the introduction of WGEP). En bloc resection was completed in 41 patients (83.7%) and adverse events occurred in 17 patients (34.7%) (Table 1).

**Table 1:** Patient characteristics.

Number of patients	Total	November 1995 to July 2005	August 2005 to April 2017	P-value
	49	16	33	
Age, median (IQR) in years	65 (59–70)	66.5 (56.5–74.8)	64 (59–69.5)	0.4
Sex, male/female	29/20	10/6	19/14	>0.99
Tumor size, median (IQR) in mm	11 (10–20)	13 (10–25)	11 (9–20)	0.31
Final histological diagnosis, n, (%)				0.08
Adenoma	33 (67.3%)	8 (50%)	25 (75.8%)	
Carcinoma-in-adenoma	6 (12.2%)	4 (25%)	2 (6.1%)	
Adenocarcinoma	8 (16.3%)	3 (18.8%)	5 (15.2%)	
Hyperplasia	1 (2%)	0	1 (3.0%)	
No tumor	1 (2%)	1 (6.3%)	0	
En bloc resection	41 (83.7%)	12 (75%)	29 (87.9%)	0.41
Additional therapy	10 (20.4%)	5 (31.3%)	5 (15.2%)	0.26
Papillectomy/APC	7 (14.3%)	4 (25%)	3 (9.1%)	0.20
Pancreatoduodenectomy	3 (6.1%)	1 (6.3%)	2 (6.1%)	>0.99
Adverse events	17 (34.7%)	7 (43.8%)	10^¶^ (30.3%)	0.52
Pancreatitis	4 (8.2%)	2 (12.5%)	2 (6.1%)	0.59
Bleeding	10 (20.4%)	4 (25%)	6 (18.2%)	0.71
Cholangitis	3 (6.1%)	1 (6.3%)	2 (6.1%)	>0.99
Peritonitis	1 (2.0%)	0	1 (3.0%)	>0.99

^¶^One patient developed cholangitis and bleeding simultaneously.

IQR, interquartile range, APC, argon plasma coagulation

**Fig 2:**
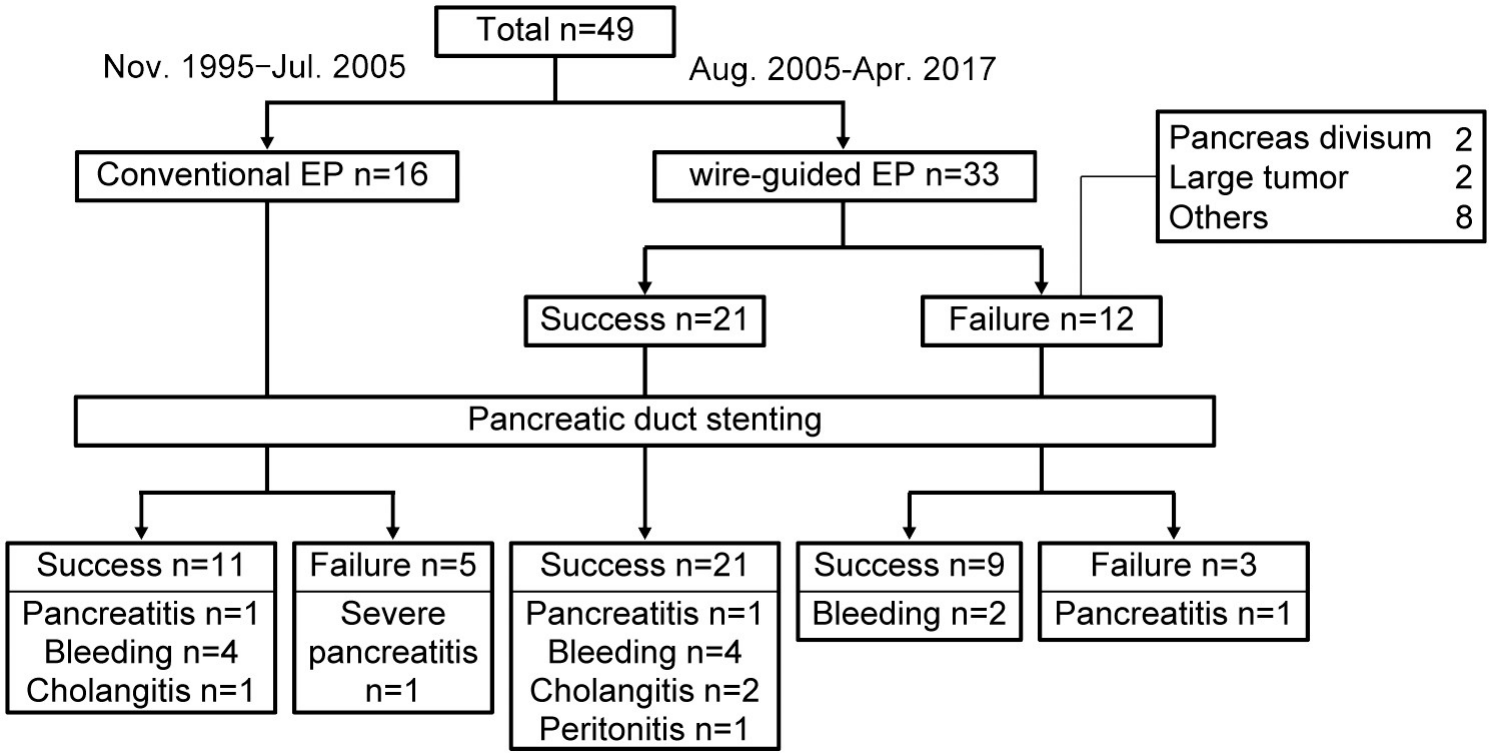
Flowchart indicating the number of participants and complications experienced for each period of time and procedure.

**Fig 3:**
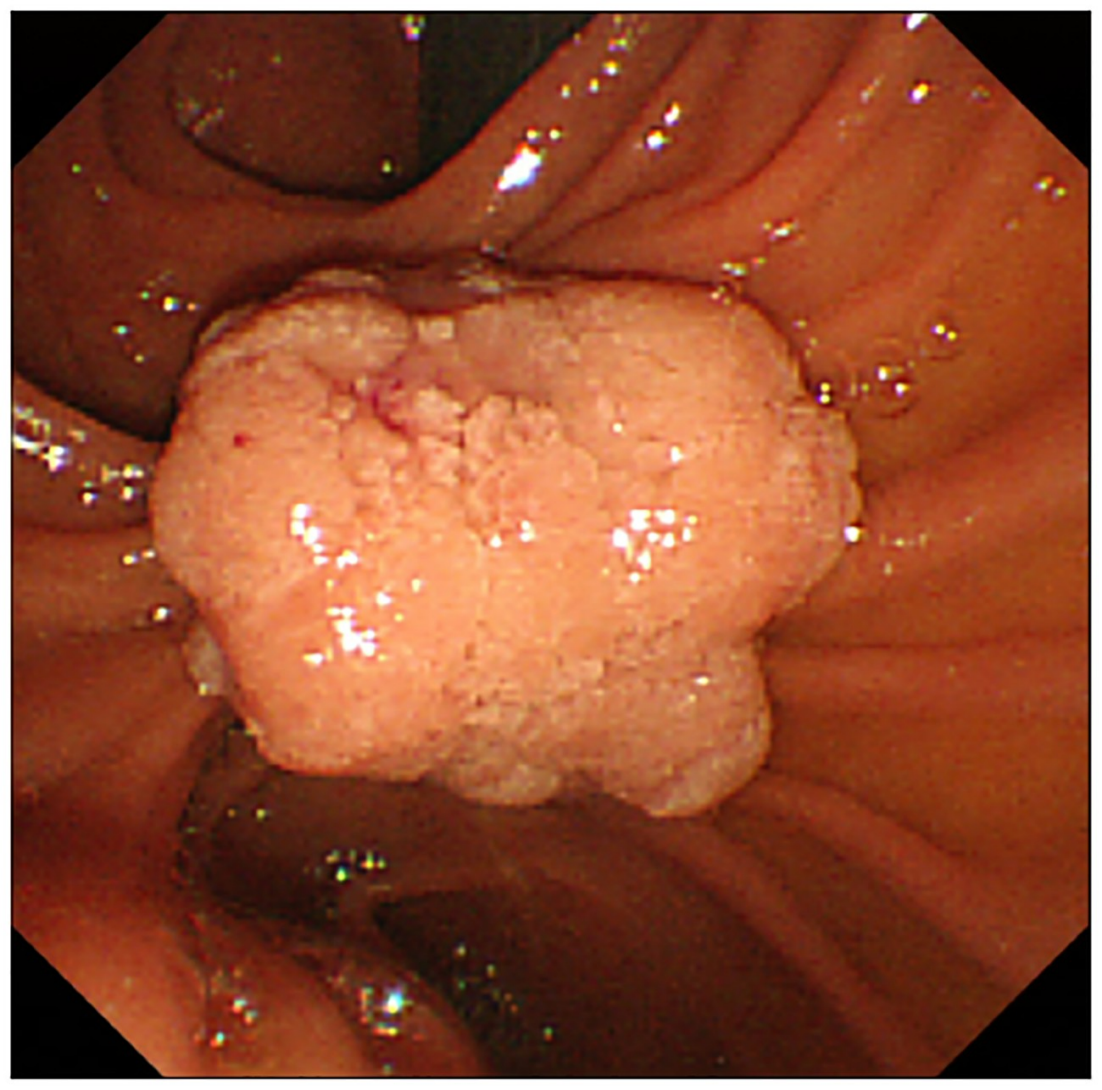
A patient in whom wire-guided endoscopic papillectomy failed due to a large-sized tumor.

**Fig 4:**
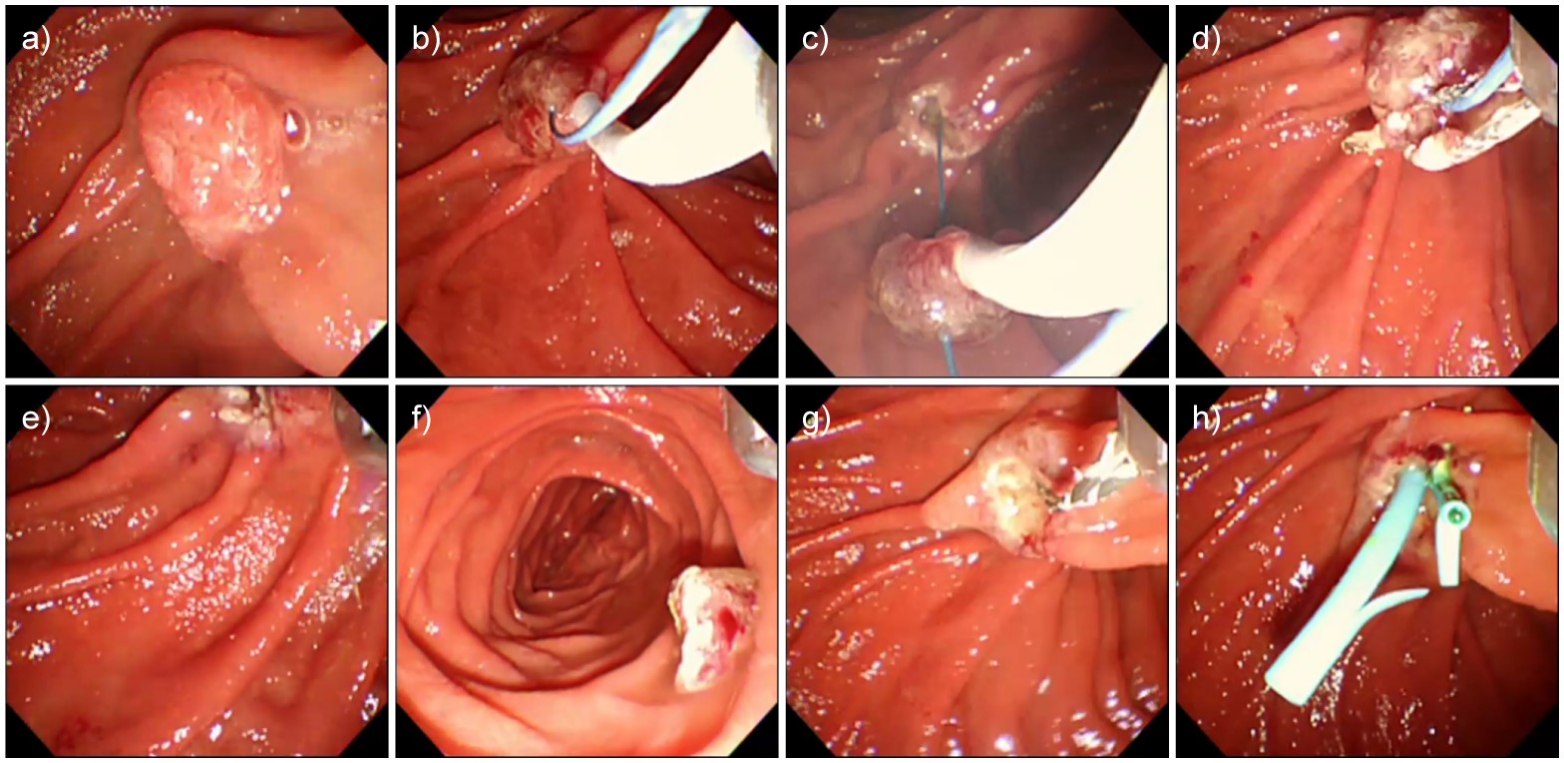
A patient in whom wire-guided endoscopic papillectomy failed due to technical trouble. a) Tumor of the papilla prior to the procedure. b) An insulated guidewire was placed into the pancreatic duct, and the papilla was grasped. c) The papilla was resected. d) The snare was detached from the specimen and removed into an endoscope. e) The tumor fell off the endoscope because the snare caught on the insulated guidewire, and then the snare and guidewire came out simultaneously. f) The fallen tumor. Using the net, we collected the tumor with the scope. g) We reinserted the scope and cannulated the pancreatic and biliary ducts. The guidewire was placed in the same manner as that used in conventional endoscopic papillectomy. h) A stent was placed into the pancreatic and biliary ducts.

We compared the data of 16 patients who underwent conventional EP between November 1995 and July 2005 and 33 patients in whom WGEP was first attempted after the introduction of this technique in August 2005. All 21 patients in whom WGEP was successfully accomplished underwent pancreatic duct stenting. However, WGEP could not be completed in 12 patients (Fig 2). Among these 12 patients, 2 had pancreas divisum and 2 had relatively large tumors that prevented prior pancreatic duct cannulation before their resection (Fig 3, S2 Video). In the other 8 patients, WGEP could not be completed because of technical difficulties such as disengagement of the guidewire from the duct during the procedure (Fig 4, S3 Video).

The rate of en bloc resection tended to be higher while the frequency of additional EP or APC was lower in the WGEP group than in the conventional EP group (87.9% vs. 75% and 9.1% vs. 25%, respectively), although no significant differences were identified. The frequency of additional pancreatoduodenectomy was similar between the WGEP and conventional EP groups (6.1% vs. 6.3%).

The incidence of cholangitis was 6.3% (1/16) in the conventional EP group and 6.1% (2/33) in the WGEP group, and no significant difference between the groups was observed. In these 3 cholangitis patients, we were able to place a bile duct stent in 1 patient in the WGEP group, while a stent could not be placed in the other 2 patients. The incidence of bleeding was 25% (4/16) in the conventional EP group and 18.2% (6/33) in the WGEP group. Although the incidence was reduced slightly in the WGEP group, no significant difference was identified. No instances of perforation were observed.

Severe acute pancreatitis occurred in 1 patient in whom pancreatic duct stenting during conventional EP could not be completed. Among the patients who underwent WGEP, acute pancreatitis occurred in 1 in whom pancreatic duct stenting was accomplished and in 1 in whom pancreatic duct stenting could not be completed; however, both cases were mild, and the patients improved after a few days (Fig 2).

**Fig 5:**
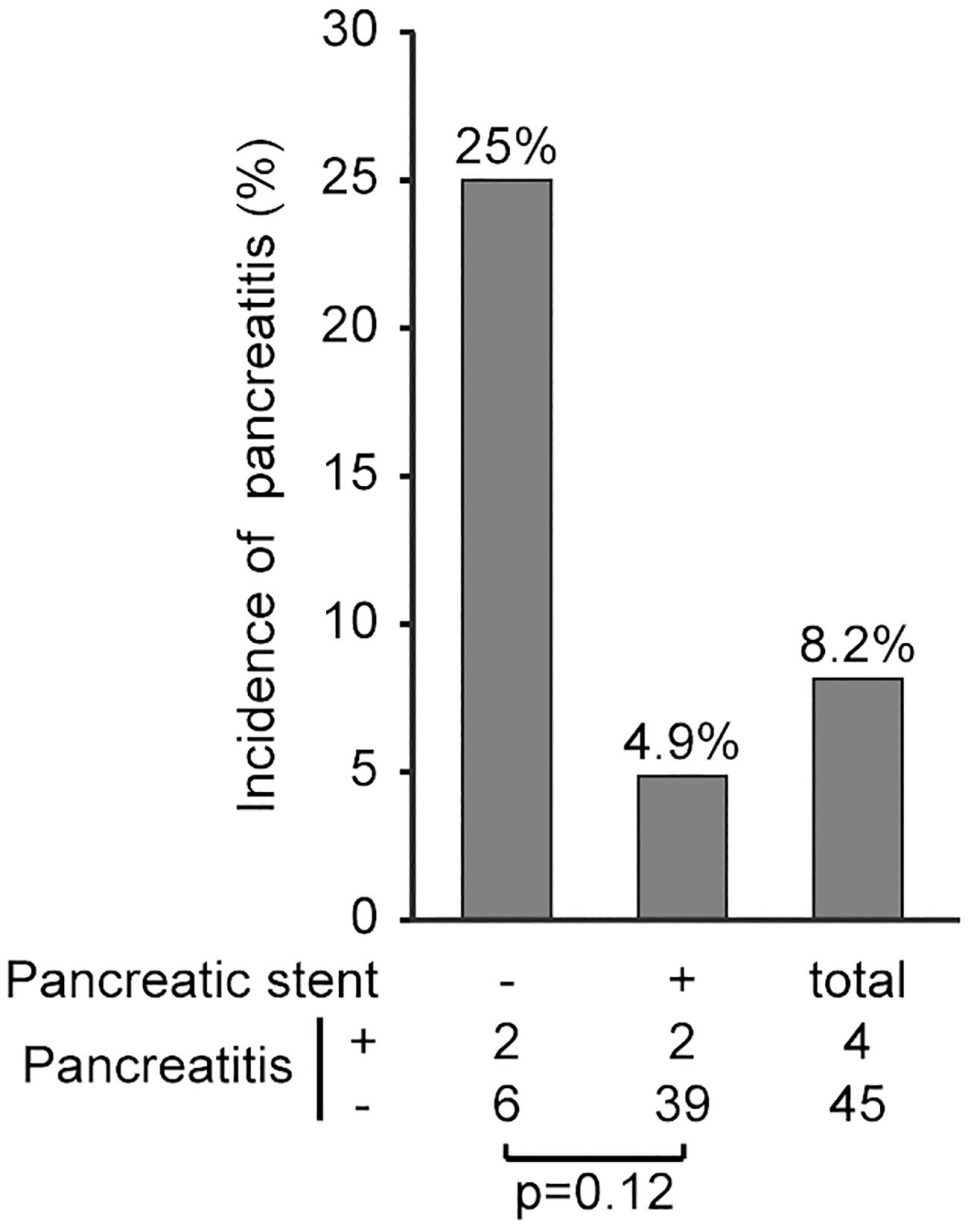
The incidence of pancreatitis according to the success or failure of pancreatic duct stenting.

Because only a few patients developed pancreatitis, there was no statistically significant difference in the prevention of pancreatitis between the groups. However, the incidence of pancreatitis was different depending on the success or failure of pancreatic duct stenting. Specifically, the incidence of post-EP pancreatitis among those patients in whom a pancreatic stent could be placed was 4.9% (2/41 patients), whereas the incidence among patients in whom a pancreatic stent could not be placed was 25% (2/8 patients), indicating that 1 in 4 of these patients developed pancreatitis (Fig 5).

**Fig 6:**
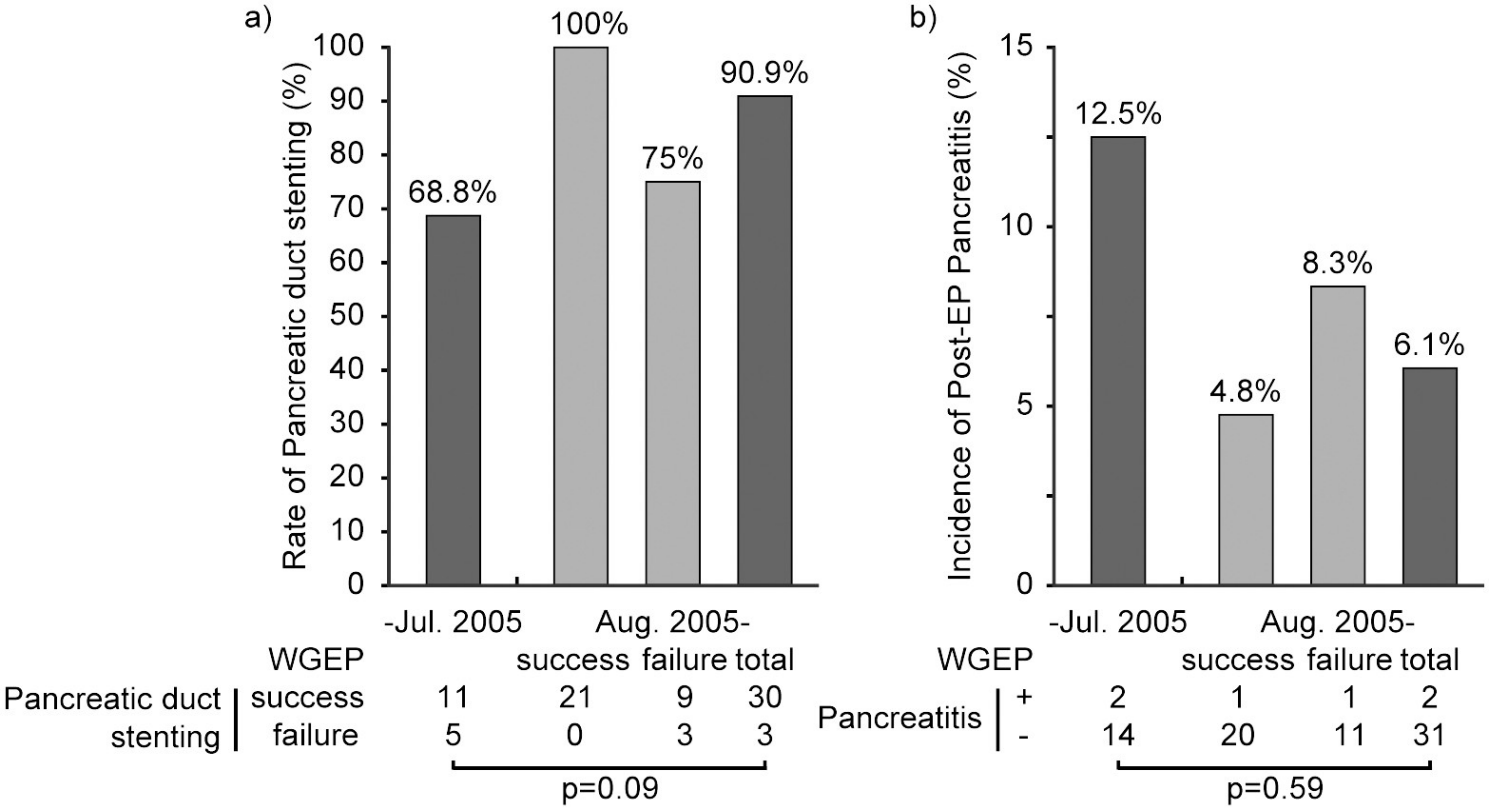
Comparisons of the rates of pancreatic duct stenting (a) and post-EP pancreatitis (b) before and after the introduction of the wire-guided method. WGEP: wire-guided endoscopic papillectomy; EP: endoscopic papillectomy.

We calculated the rate of pancreatic duct stenting before and after the introduction of WGEP. Because pancreatic duct stenting is definitely possible if WGEP is completed, after introducing WGEP, pancreatic duct stenting was possible in 30 of the 33 patients in whom WGEP was attempted (90.9%); this rate was much higher than that before the introduction of WGEP (Fig 6a). Because only a few patients developed pancreatitis, the incidence of pancreatitis was not significantly different between the groups; however, the incidence of pancreatitis was reduced by half from 12.5% (2/16 patients) to 6.1% (2/33 patients) after the introduction of WGEP (Fig 6b).

## Discussion

The present study retrospectively analyzed the utility of WGEP versus conventional EP by comparing the rates of pancreatic duct stenting and pancreatitis before and after the introduction of WGEP at our institution.

Suzuki et al. [14] first performed EP in 1983, and today, it is considered an effective treatment for duodenal papillary adenomas and some papillary carcinomas [2]. On the other hand, EP is associated with a high rate of complications. Hemostasis after bleeding and suture perforation require the endoscopist to have a high level of experience and skill, and postoperative complications, such as acute pancreatitis and cholangitis, require intensive care [15]. Particularly in patients with pancreatitis, death has been reported [3], but, since many of the diseases being treated were benign, deaths due to complications cannot be tolerated. Therefore, preventive methods need to be developed.

The WGEP procedure we employed in this study was developed by Moon et al. [12] in 2005. In this study, we employed a modified WGEP method, which used a smaller caliber guidewire. Although this guidewire is very soft and cannot be used for stent placement, it has good flexibility, which allowed us to easily control the scope during the snaring and resection of the tumor.

Although the WGEP procedure was expected to be useful for preventing post-EP pancreatitis [16], recent reports suggest that it does not lower the incidence of post-EP pancreatitis [17]. However, if pancreatic duct stenting can prevent pancreatitis, it is conceivable that WGEP, in which pancreatic stent placement is certain, will effectively prevent pancreatitis. Our data confirm that pancreatic duct stenting clearly reduces the incidence of pancreatitis (Fig 5). Moreover, we examined the incidence of post-EP pancreatitis after WGEP was introduced at our institution. Here, pancreatic duct stenting was successful in all 21 of the patients in whom WGEP could be completed, and the incidence of pancreatitis was very low (4.8%). When pancreatitis occurred, its severity was mild. Even if we included those patients in whom WGEP could not be completed, the incidence was still reduced by half, from 12.5% before the introduction of WGEP to 6.1% thereafter. Both in the conventional EP group and in the WGEP-failed group, pancreatic duct stenting was successful in only three fourths of the patients. According to this rate, in the group where WGEP succeeded, if WGEP had not been attempted, pancreatic duct stenting might not have been completed in five or six patients, and of these patients without a pancreatic stent, one or two patients might have developed pancreatitis. This result suggests that WGEP shows some potential for reducing the total incidence of post-EP pancreatitis owing to more successful pancreatic duct stenting, even when we include those patients in whom the procedure could not be completed.

Herein, despite the lack of a significant difference, the rate of en bloc resection tended to higher and the frequency of additional EP or APC was lower in the WGEP vs. conventional EP group. As preservation of the pancreatic duct might enable the operator to feel sufficiently secure to perform EP and achieve reliable manipulation, this may partially explain the higher en bloc resection rate and lower additional therapy rate. This result suggests that WGEP may raise the rate of complete resection. Additional studies with more patients are needed to evaluate this aspect in more detail.

Our study has several important limitations. First, because this was a retrospective study and only a few patients developed post-EP pancreatitis, the statistical analyses were limited, and significant differences could not be confirmed. A prospective study is needed to obtain more concrete results. Second, although WGEP is reported to be a complicated and long procedure with a low rate of complete resection, we did not evaluate the procedure time and histological complete resection. Moreover, in the present study, we observed eight patients in whom WGEP could not be completed owing to technical problems. As such, technique improvements and methods for shortening the procedure time are needed. Nevertheless, WGEP has been shown to be safe and useful for reducing the incidence of pancreatitis, thus we believe it is worth attempting. Finally, it is known that endoscopists must have a high skill level to perform EP. Indeed, the frequency of complications is related more to the skill of the operator and to the method employed than it is to the device used. Because ours was a long-term study, it is possible that the results were influenced by the experience of the operator; in addition, the device used changed throughout the study. However, the operators were already experts at the time of study initiation, and the devices did not change extensively. It is unlikely that the change affected the incidence of post-EP pancreatitis. Therefore, we believe that our results still provide valuable information regarding the utility of WGEP versus conventional EP.

In conclusion, the present study showed that successful WGEP can facilitate pancreatic stenting. We also demonstrated that by using this procedure, it is possible to reduce the number of patients in whom a pancreatic stent cannot be placed, which in turn may reduce the overall incidence of pancreatitis. We believe that WGEP is a safe method that is worth attempting, as it shows some potential for preventing post-EP pancreatitis.

## Supporting information

S1 Video Pancreatic duct wire-guided endoscopic papillectomy. (MP4)

S2 Video A patient in whom wire-guided endoscopic papillectomy failed due to a large-sized tumor. (MP4)

S3 Video A patient in whom wire-guided endoscopic papillectomy failed due to technical trouble. (MP4)
